# A Rare Diagnosis of Caroli Syndrome in a Young Patient

**DOI:** 10.1002/ccr3.70555

**Published:** 2025-05-30

**Authors:** Elaheh Karimzadeh‐Soureshjani, Farab Pourhasan, Pouria Ahmadi Simab, Sajjad Shojaei, Yasaman Mohammadian

**Affiliations:** ^1^ Department of Clinical Sciences Abadan University of Medical Sciences Abadan Iran; ^2^ Student Research Committee Abadan University of Medical Sciences Abadan Iran; ^3^ Universal Scientific Education and Research Network (USERN) Tehran Iran; ^4^ Department of Radiology Taleghani Hospital, Abadan University of Medical Sciences Abadan Iran; ^5^ Abadan University of Medical Sciences Abadan Iran

**Keywords:** autosomal recessive polycystic kidney disease, Caroli syndrome, liver cysts, medullary sponge kidney, MRCP

## Abstract

Caroli syndrome is a rare but serious congenital disorder associated with portal hypertension and polycystic kidney disease. Early diagnosis via imaging, particularly MRCP, is crucial to prevent life‐threatening complications such as cholangitis and biliary cirrhosis. Timely intervention and close monitoring can significantly improve patient outcomes.

**Summary**

- Early diagnosis of Caroli syndrome requires high clinical suspicion even without classic manifestations like cholangitis or ARPKD.
- Ultrasound can be a valuable diagnostic tool in resource‐limited settings by detecting signs like the “dot sign.”
- Long‐term follow‐up and management of complications like portal hypertension and cholangiocarcinoma are crucial.

## 1 Introduction

Caroli disease was first described in 1958 by Dr. Jacques Caroli, a French gastroenterologist who observed “multifocal, non‐obstructive, saccular or fusiform segmental dilatation of the intrahepatic bile ducts” [1]. Caroli disease is a congenital condition defined by multifocal, segmental dilation of the large intrahepatic bile ducts. It is often linked with cystic kidney disease, which can vary in severity. Caroli identified two distinct types of bile duct dilation: Caroli disease, which involves bile duct ectasia without additional liver abnormalities, and Caroli syndrome, where, in addition to ductal dilation, congenital hepatic fibrosis is also present.

The incidence of Caroli syndrome is higher than that of Caroli disease [2] (1/1,000,000 for Caroli disease, compared with 1/100,000 for Caroli syndrome [3]). Men and women are affected equally, and it is more common in people of Asian descent [1] Symptoms of Caroli syndrome typically emerge in early adulthood, with over 80% of cases manifesting by the age of 30. Patients often present with fever, jaundice, and abdominal pain, commonly resulting from cholangitis. Individuals with Caroli syndrome may also exhibit signs of acute cholangitis or non‐cirrhotic portal hypertension along with its associated complications. Upon physical examination, the liver is frequently enlarged, and splenomegaly is observed due to portal hypertension [3]. The diagnosis is confirmed through the detection of cystic dilation in the proximal intrahepatic bile ducts, while the common bile duct remains normal, in patients presenting with acute cholangitis or elevated liver function tests. This can be observed via ultrasonography or Magnetic Resonance Cholangiopancreatography (MRCP) [3]. Ultrasonography is the best initial measure because it is inexpensive, rapid, and noninvasive. Although it has low specificity and is operator‐dependent, it may show irregular dilatation of the intrahepatic bile ducts, sometimes accompanied by dilatation of the extrahepatic ducts due to cholestasis [4]. This case report aims to present the clinical, radiological, and laboratory findings of Caroli syndrome in a young male patient, emphasizing its diagnosis, complications, and the importance of early intervention.

## 2 Case History and Examination

A 16‐year‐old male presented to a university‐affiliated hospital in Abadan, Iran, in June 2024 with abdominal pain and fever. The pain was primarily localized to the right upper quadrant (RUQ), radiating to the back, and described as vague and progressive over the past 3 days, without relief from positional changes. The patient reported intermittent nighttime fever, but he was afebrile upon admission (36.4°C). Additional symptoms included mild nausea without vomiting, weight loss, melena, or hematochezia. He denied dyspnea, and the pain was unrelated to physical activity.

On physical examination, RUQ tenderness and guarding were noted, though abdominal distension was absent. Splenomegaly was detected, but there were no changes in urine or stool color. The patient reported similar episodes occurring repeatedly over the past few months. His medical history was unremarkable except for prior antibiotic use (unknown names). He denied alcohol consumption and had no significant family history of liver or biliary diseases.

### 2.1 Differential Diagnosis, Investigations, and Treatment

Initial laboratory investigations revealed pancytopenia, including leukopenia, thrombocytopenia, and low hemoglobin levels, while bilirubin and liver enzyme levels remained within normal limits (Table 1). Given the patient's symptoms and laboratory abnormalities, an abdominal and pelvic ultrasound was performed, which demonstrated a coarse echogenic liver with left lobe hypertrophy and right lobe atrophy. Additionally, there was increased gallbladder wall thickness, while the Common Bile Duct (CBD) and extrahepatic bile ducts appeared normal. Notably, cystic dilatation of the intrahepatic bile ducts was observed, with the presence of the “dot sign,” strongly suggestive of Caroli disease.

**TABLE 1:** Laboratory profile of a 16‐year‐old patient with Caroli syndrome.

Test	Result	Unit	Normal range
WBC	1160	cells/μL	4000–10,000
Hb	8.5	g/dL	14–18
Platelet	73	10 × 3/μL	130–400
SGOT	15	IU/L	0–37
SGPT	6	IU/L	0–41
ALKP	209	U/L	80–306
Total bilirubin	0.5	mg/dL	0.1–1.2
Direct bilirubin	0.3	mg/dL	0.1–0.3
Na	138	mmol/L	135–145
K	3.8	mmol/L	3.5–5.5
Amylase	57	U/L	24–100
Lipase	49	U/L	13–60
BUN	19	mg/dL	8–20
Cr	1.2	mg/dL	0.7–1.4

Abbreviations: ALKP, alkaline phosphatase; BUN, blood urea nitrogen; Cr, creatinine; Hb, hemoglobin; K, potassium; Na, sodium, SGOT, serum glutamic oxaloacetic transaminase; SGPT, serum glutamic pyruvic transaminase; WBC, white blood cells.

**FIGURE 1:**
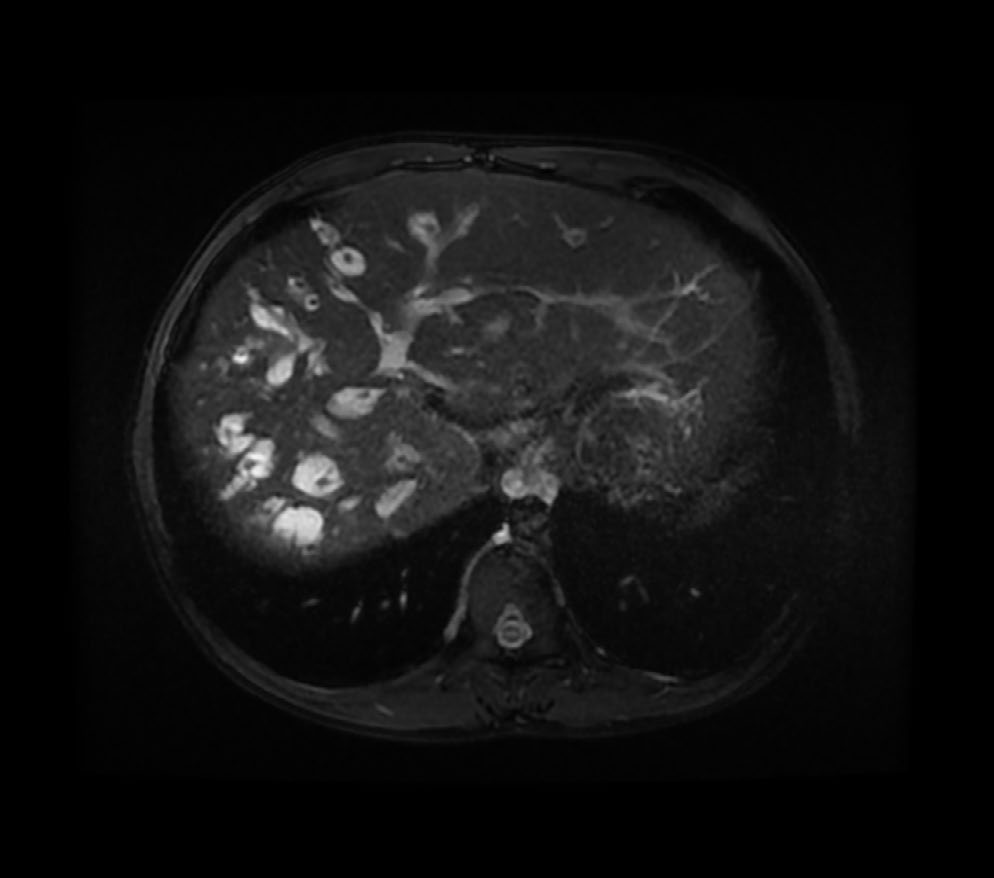
Multiple cystic dilatations of the biliary tree with portal vein branches at the center, observed on MRCP, suggestive of polycystic liver disease. This imaging finding indicates a significant involvement of the biliary system, often associated with systemic polycystic disease.

Further Doppler ultrasound assessment of hepatic vasculature revealed an elevated End‐Diastolic Velocity (EDV) of the hepatic artery relative to the Peak Systolic Velocity (PSV) of the portal vein, consistent with portal hypertension. The spleen was significantly enlarged (212 mm), and multiple varicose veins with splenorenal connections were observed. Additionally, the kidneys were enlarged with increased echogenicity and medullary calcifications, findings suggestive of medullary sponge kidney.

**FIGURE 2:**
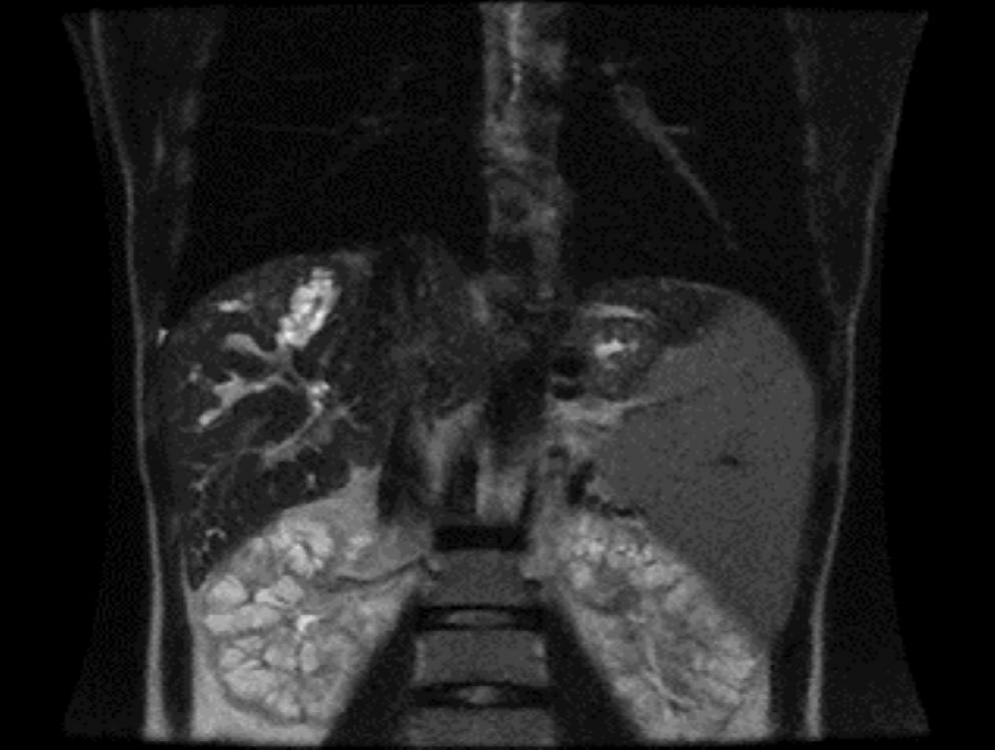
Typical manifestations of polycystic kidney disease observed on MRCP, showing multiple renal cysts that are characteristic of this genetic disorder. This imaging feature assists in diagnosing polycystic kidney disease, which can lead to renal enlargement and dysfunction over time.

To confirm the diagnosis, Magnetic Resonance Cholangiopancreatography (MRCP) was performed, revealing an enlarged liver with multiple cystic ectasias affecting the intrahepatic bile ducts, while the extrahepatic bile ducts remained normal (Figure 1). The presence of portal hypertension and Autosomal Recessive Polycystic Kidney Disease (ARPKD) further supported the diagnosis of Caroli syndrome (Figure 2).

### 2.2 Follow‐Up and Outcome

The patient was advised to undergo long‐term follow‐up and supportive management, including monitoring for portal hypertension and progressive liver dysfunction. Potential future interventions, such as liver transplantation in advanced cases, were discussed. Despite the need for close monitoring and further evaluation, the patient declined further treatment, making follow‐up impossible.

## 3 Discussion

The cause of Caroli disease remains unknown, but genetic profiling seems to play a role. It is believed to be autosomal recessive in Caroli syndrome and sporadic in Caroli disease. Mutations studied so far involve genes that are crucial for the development of the kidneys and the biliary tree [5]. Caroli syndrome is linked to autosomal recessive polycystic kidney disease (ARPKD), which results from pathogenic variants in the PKHD1 gene. This gene encodes fibrocystin, a large membrane protein predominantly expressed in the kidneys, with lesser expression in the liver. Pathogenic mutations in the PKD1 or PKD2 genes are associated with autosomal dominant polycystic kidney disease (ADPKD), which can occasionally co‐occur with Caroli disease. Additionally, mutations in the WDR19 gene cause nephronophthisis (NPHP), a disorder that frequently progresses to end‐stage kidney disease (ESKD) and may be associated with Caroli syndrome or disease. NPHP arises from mutations in various genes that encode proteins crucial for the function of fibrils, basal bodies, and centrosomes, leading to renal dysfunction and extra‐renal manifestations, including retinal degeneration, cerebellar ataxia, and hepatic fibrosis [6]. The clinical features of Caroli disease typically include recurrent episodes of cholangitis, gallstones, biliary abscesses, and septicemia. Chronic liver failure and portal hypertension due to hepatic fibrosis may also be present. Laboratory findings are generally nonspecific [7]. Leukocytosis and elevated transaminase levels are common in cholangitis [8]. Splenomegaly or cytopenia due to hypersplenism may suggest portal hypertension, as seen in our patient [7].

Most patients with Caroli syndrome (CS) are not diagnosed until complications of portal hypertension have developed. Some patients may test positive for autoantibodies, which can be misleading and lead to a misdiagnosis. Although ultrasound (US) is not ideally accurate, the presence of liver cysts and splenomegaly detected through ultrasound may raise initial suspicion for CS. Early suspicion of the disease is likely the most critical factor in reducing the diagnostic delay in Caroli syndrome [9].

Complications of Caroli syndrome (CS) include cholangitis, sepsis, choledocholithiasis, liver abscess, cholangiocarcinoma, and portal hypertension [7]. Approximately 30% of individuals with Caroli syndrome develop intrahepatic stones. These stones are a contributing factor to recurrent episodes of cholangitis, which can eventually result in secondary biliary cirrhosis [3]. A few studies have investigated the association between CS, cystic disease (CD), and cancer; however, most of these studies do not offer a clear explanation for this correlation. The overall incidence of malignant or concurrent cancer in patients with CS and CD is around 7%, though it ranges from approximately 3%–40%. No new findings regarding the etiology of malignant transformation have been reported [10]. It seems that the development of cancer in Caroli disease is linked to biliary stasis, the action of carcinogens in bile, and chronic inflammation of the biliary epithelium, which leads to dysplastic changes in the tissue [11]. Most cases of cancer in Caroli disease are cholangiocarcinoma or adenocarcinoma, with the biliary epithelium serving as the source of the cancerous transformation [10]. Additionally, amyloidosis has been reported as another complication of Caroli disease [11].

**FIGURE 3:**
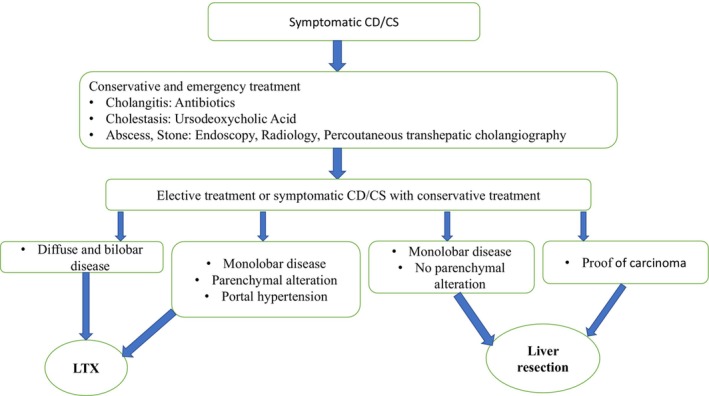
Algorithm for the treatment of symptomatic Caroli disease (CD) or Caroli syndrome (CS) with liver resection or liver transplantation (LTX) [14].

While Caroli syndrome is well‐described, our case highlights unique diagnostic and management challenges when contextualized with key literature. Similar to Akhan et al.'s [12] cohort, where 78% of patients presented with portal hypertension, our patient exhibited splenomegaly and varices, yet differed in lacking cholangitis a feature present in 82% of cases. In contrast to Wang et al. [13], who reported choledocholithiasis in 60% of adolescents with Caroli syndrome, our case showed no biliary stones, reinforcing the disease's phenotypic variability. Notably, the medullary sponge kidney finding in our patient diverges from the classic ARPKD association in Gunay‐Aygun et al. [14], suggesting broader renal manifestations. These distinctions underscore the need for vigilance in atypical presentations, particularly in resource‐limited settings where MRCP access is constrained (Figure 3).

Treatment for Caroli syndrome is primarily supportive and requires a tailored approach for each patient [15]. The management of acute cholangitis in patients with Caroli syndrome generally involves supportive care, the administration of antibiotics, and biliary drainage [3]. The differential diagnosis of Caroli syndrome includes several conditions that can present with similar symptoms. These conditions include chronic hepatitis, liver cirrhosis, gallstones, and certain genetic disorders such as polycystic kidney disease and cystic fibrosis. Caroli syndrome, in particular, shares similarities with biliary diseases that cause obstruction and increased pressure in the biliary system, such as chronic cholangitis. Additionally, hepatic abnormalities like cirrhosis can present with symptoms similar to Caroli syndrome, including splenomegaly and portal hypertension. Therefore, precise diagnostic tests such as liver ultrasound and MRCP are essential for distinguishing these conditions from one another [16]. The choice of surgical intervention depends on the degree of biliary dilatation and the extent of liver parenchymal damage, such as fibrosis or cirrhosis. Surgical treatment should be initiated promptly to prevent life‐threatening complications and reduce the risk of carcinogenesis. For monolobar disease without liver parenchymal changes, local liver resection is recommended. In patients with diffuse, bilateral disease involving significant parenchymal and functional alterations, liver transplantation (LTX) should be considered (Figure 3) [17]. Additionally, the management of Caroli syndrome should include strategies for the prevention and treatment of complications associated with portal hypertension [3]. While Caroli syndrome remains a well‐described entity, this case underscores critical clinical lessons: first, the diagnostic challenge of atypical presentations (e.g., predominant portal hypertension with subtle renal involvement), emphasizing the need for heightened suspicion even without classic ARPKD; second, the pragmatic role of ultrasound “dot sign” in resource‐limited settings, despite MRCP being gold‐standard; and third the ethical and logistical hurdles in managing young, non‐adherent patients a scenario rarely addressed in literature. These observations call for standardized regional guidelines to address genetic testing gaps (e.g., PKHD1 in Middle Eastern populations) and multidisciplinary frameworks to improve long‐term follow‐up.

## 4 Conclusion

Caroli syndrome is an uncommon genetic condition marked by the dilation of intrahepatic bile ducts and is frequently associated with polycystic kidney disease. Early diagnosis is crucial to prevent complications such as cholangitis, sepsis, and portal hypertension. Imaging techniques, particularly MRCP, play a vital role in confirming the diagnosis and differentiating it from other biliary and hepatic disorders. The management of Caroli syndrome is primarily supportive, with treatment focusing on controlling cholangitis and preventing further liver damage. Surgical intervention, including liver resection or liver transplantation, may be required in severe cases. Awareness of this condition and a high index of suspicion are key in improving patient outcomes and minimizing delays in diagnosis.

## Author Contributions

**Elaheh Karimzadeh‐Soureshjani:** conceptualization, investigation, methodology, writing – original draft, writing – review and editing. **Farab Pourhasan:** conceptualization, investigation, methodology, writing – original draft. **Pouria Ahmadi Simab:** writing – original draft. **Sajjad Shojaei:** data curation, investigation, methodology. **Yasaman Mohammadian:** methodology, supervision.

## Ethics Statement

Ethical approval for this study was obtained from the Research Ethics Committee of Abadan University of Medical Sciences (IR.ABADANUMS.REC.1403.157). The study was conducted in accordance with the university's guidelines, ensuring the protection of patient rights and confidentiality. Informed consent was obtained from the patient's parent or their legal guardian, including consent for publication of the data in this article. The study was conducted in compliance with the ethical standards outlined in the 1964 Declaration of Helsinki and its subsequent amendments.

## Consent

Informed consent for publication of the data and the use of the patient's information in this article was obtained from the patient or their legal guardian. The consent form was signed by the patient's parent, who is the legal representative of the patient. The patient's identity has been kept confidential, and no personally identifiable information, including names, initials, addresses, or any other data that could identify the patient, is disclosed in the manuscript. All subjects gave their informed consent prior to their inclusion in the study, and any information that could reveal the identity of the patient was not published.

## Conflicts of Interest

The authors declare no conflicts of interest.

## Data Availability

The data that support the findings of this study are available from the corresponding author upon reasonable request.
